# Recombinant Avian β-Defensin Produced by Food-Grade *Lactococcus* as a Novel and Potent Immunological Enhancer Adjuvant for Avian Vaccine

**DOI:** 10.1007/s12602-021-09847-8

**Published:** 2021-09-30

**Authors:** Tingting Wang, Zhihao Wang, Jielan Mi, Wenqian Wang, Kai Li, Xiaole Qi, Yulong Gao, Li Gao, Changjun Liu, Yanping Zhang, Qing Pan, Xiaomei Wang, Hongyu Cui

**Affiliations:** State Key Laboratory of Veterinary Biotechnology, Harbin Veterinary Research Institute, Chinese Academy of Agricultural Sciences, Harbin, 150069 China

**Keywords:** Chicken β-defensin, Recombinant *Lactococcus lactis*, Immunomodulatory activity immune adjuvant

## Abstract

In this study, we expressed rAvBD1-2–6-13 protein through *Lactococcus lactis* NZ3900, and the effects of the recombinant *L. lactis* NZ3900 as an immune enhancer and immune adjuvant were verified using in vivo and in vitro tests. In vitro tests revealed that recombinant *L. lactis* NZ3900 significantly activated the NF-κB signaling pathway and IRF signaling pathway in J774-Dual™ report cells and significantly increased the transcript levels of IL-10, IL-12p70, CD80, and CD86 in chicken PBMCs and chicken HD11 cells. In vivo experiments revealed that the immunized group supplemented with recombinant *L. lactis* NZ3900 as an adjuvant had significantly higher serum antibody titers and higher proliferative activity of PBMCs in the blood of the chickens immunized with NDV live and inactivated vaccines. Our study shows that the recombinant *L. lactis* NZ3900 has strong immunomodulatory activity both in vivo and in vitro and is a potential immune enhancer. Our work lays the foundation for the research and development of new animal immune enhancers for application in the poultry industry.

## Introduction

Immune enhancers are commonly used to stimulate the immune response to the vaccines and include active immunostimulants, carriers, and vehicle adjuvants. Among these, adjuvants belong to different classes of compounds, such as alum salts and other mineral adjuvants, bacterial derivatives, vehicles, and cytokines. However, many adjuvants pose challenges such as induction of physiological disorders, anaphylactic reactions, hypersensitization, and toxicity [1]. Biosafety and green food safety put forward higher requirements for future biological agents, especially preventive biological agents. New types of vaccines, immune adjuvants, and immune enhancers must meet the basic requirements for green food safety. Defensins are a group of small cationic peptides that play an important role in the vertebrates innate immune system, as well as in invertebrates [2–4]. Beta-defensins can be produced by phagocytic cells and lymphocytes to initiate, mobilize, and enhance adaptive immune host defense [5]. Studies have shown that defensins can enhance macrophage activity, phagocytosis of chemotaxis monocytes, T lymphocytes, mast cells, and immature dendritic cells and induce the production of pro-inflammatory cytokines and immunoregulatory cytokines [6]. Βeta-defensin (β-defensin) exhibits anti-bacterial activity and has strong potential as an immune adjuvant [7] and, thus, can significantly improve the immune efficacy of the vaccine. Given that most vaccines, especially subunit vaccines, need an adjuvant for inducing effective immune response via activating innate immunity, β-defensin is a candidate stimulant of innate immunity and adaptive immune responses [8]. Avian β-defensins (AvBDs) represent a group of innate immune molecules with broad anti-microbial activity. Previous studies have identified 14 AvBDs encoded by genes in a cluster on chromosome three of the chicken genome [9].

Lactic acid bacteria (LAB) are gram-positive bacteria that produce lactic acid via carbohydrate fermentation. Most LAB species benefit animals, plants, and humans. For thousands of years, LAB have been widely and successfully used for food fermentation [10, 11]. Probiotics enhance non-specific immune functions, including the phagocytic activity of innate immune cells and the cytotoxicity of NK cells [12]. Probiotics stimulate the body to produce cytokines, stimulate B cells to secrete IgA and IgG, inhibit IgE production, and activate helper T lymphocytes and macrophages [13, 14]. Cell wall, nucleic acids, and other components of LAB exhibit adjuvant activity to certain extent. Among the many benefits of probiotics, their ability to regulate the immune system has received the most attention. Some studies have provided clear evidence that certain probiotics can induce polarization of the immune response by stimulating the release of specific cytokines by activated dendritic cells. Many studies have demonstrated the immune system modulatory properties of probiotics, including the activation of the immune system and downstream immune responses [15].

Recombinant LAB can be used as live vectors for the expression and delivery of therapeutic and preventive drugs [5, 16]. Although expression of bird β-defensin using an *Escherichia coli* expression system has been reported [17], the expressed products exist in the form of inclusion bodies. The process of refolding AvBDs from inclusion bodies to form biologically active AvBDs is complicated, and a variety of endotoxins also exist [11, 18, 19]. In this study, LAB and food-grade expression vectors were used to construct recombinant *Lactococcus lactis* NZ3900 expressing chicken rAvBD1-2–6-13, and the immunomodulatory activities of expressed recombinant avian defensins were evaluated. The recombinant avian beta-defensin rAvBD1-2–6-13 showed good potential as a vaccine adjuvant, and its development as a green food safety grade vaccine adjuvant or immune booster should be explored further.

## Materials and Methods

### Bacterial Strains, Cells, and Growth Conditions

The bacterial strain used in this study was *L. lactis* NZ3900 (MoBiTec, Gottingen, Germany); *L. lactis* NZ3900 competent cells were grown in GEM medium (0.5% glucose Elliker medium) at 30 °C without agitation, and 10 μg/mL chloramphenicol was added as necessary. The plasmid used in this study was pNZ8149-nisKR-Cm, which is named pNZ81489t. In the LAB strain *L. lactis* NZ3900, the nisK and nisR genes were inserted into the pepN gene site, as the plasmid that contains these genes is more conducive to the induction of recombinant protein expression. *L. lactis* NZ3900 is a recipient bacterium lacking the chloramphenicol resistance gene (Cm) that cannot be grown in chloramphenicol-containing medium (selective medium). Thus, the strain can grow only when the vector carrying the chloramphenicol resistance gene enters the cell.

Peripheral blood mononuclear cells (PBMCs) were isolated from 6- to 11-week-old SPF chicken using a chicken peripheral blood lymphocyte separation solution kit (TBD, China). PBMCs were collected, washed in RPMI 1640 medium (Sigma, USA), and adjusted to 1 × 10^6^ cells/mL in RPMI1640 supplemented with gentamicin (150 μg/mL), 2 mM L-glutamine, and 10% fetal bovine serum (FBS) (Gibco, USA).

The macrophage cell line J774-Dual™ (InvivoGen, Hong Kong) was cultured in DMEM (Sigma, USA) medium supplemented with 10% fetal bovine serum, 100 mg/mL Normocin, 100 U/mL penicillin, 100 μg/mL streptomycin, 5 µg/mL blasticidin, and 100 µg/L Zeocin, adjusted to 2.8 × 10^5^ cells/mL in 5% CO_2_ and 37 °C atmosphere. J774-Dual™ cells were derived from the mouse macrophage-like cell line J774.1 by stable integration of two inducible reporter constructs. J774-Dual™ cells allow the simultaneous study of the NF-kB pathway by assessing the activity of SEAP and the interferon regulatory factor (IRF) pathway, by monitoring the activity of Lucia luciferase. LPS, 2′3′-cGAMP, and Pam3CSK4 were purchased from InvivoGen Co., Ltd., Hong Kong.

The chicken macrophage cell line HD11 was cultured to a concentration of 1 × 10^6^ cells/mL in DMEM medium supplemented with 10% fetal bovine serum, 2 mM L-glutamine, 100 U/m L penicillin, and 100 μg/mL streptomycin at 37 °C and 5% CO_2_.

A DNA gel extraction and purification kit was purchased from Axygen. One Step Cloning Kit was purchased from Vazyme; Primer Star Max, SYBR Green Realtime PCR Master Mix, and Premix Ex Taq (Probe qPCR) kits were purchased from Toyobo Biotechnology Co., Ltd.; Ex Taq enzyme, RNAiso Plus is a product of TaKaRa; HA mouse monoclonal antibody was purchased from Beijing Boaolong Company; and Newcastle disease oil emulsion inactivated vaccine, Newcastle disease live (NDV) vaccine (Lasota strain), and NDV virulent strain (F48E9) were purchased from Harbin Vike Biological Co., Ltd. Cell Counting Kit-8 (purchased from Dojindo, Japan).

### DNA Manipulation and Recombinant Plasmid Construction

According to the avian β-defensin (AvBD1, AvBD2, AvBD6, and AvBD13) gene sequences obtained by GenBank, the secreted signal peptide sequences were removed, and the remaining four functional segments using the linker gene encoding (G4S)3, spliced into AvBD1 (G4S)3, AvBD2 (G4S)3, AvBD6 (G4S)3AvBD13 segment (named AvBD1-2–6-13), and AvBD1-2–6-13 segments were genetically optimized and synthesized by Genescript Biotech based on the genome of the host strain *L. lactis* NZ3900. Synthesized optimized rAvBD1-2–6-13 was amplified using the forward primer 5′-ATGGGTACTGCAGGCATGCTTGGAGGTGGAGGTTCAGGTCGTAAATCAGATTGTTTTCGTAAA-3′ and the reverse primer 5′-CTCTCTAGAACTAGTGGTACCTTAAGCGTAATCTGGAACATCGTATGGGTAAGTTCCACCAAGTGAAGGAGATGGATCTTGTTCAGCTGTATGAAG-3 and cloned into the plasmid pNZ8149t by homologous recombination using the One-Step Cloning kit (Vazyme Biotech Co., Nanjing, China). Cloned regions were sequenced after each stage of construction, and the final recombinant plasmid was electro-transformed into competent *L. lactis* NZ3900. r-*L. lactis*-AvBD1-2–6-13 was selected on Elliker culture agar plates supplemented with 0.5% lactose and 10 μg/mL chloramphenicol and then grown at 30 °C according to standard protocols [20].

### Nisin-Controlled Expression, Protein Extraction, and Western Blotting Analysis

Recombination *Lactococcus* was inoculated in L-Elliker liquid medium at a 1:100 ratio. When the OD value was 0.35, the inducer nisin was added to a final concentration of 10 ng/mL, incubated in a 30 °C incubator for 6 h, and washed twice with PBS 10 times more concentrated than the bacterial solution. An ultrasonic disruptor was used for ultrasonic disruption, followed by centrifugation at 3000 g for 10 min. The supernatant was collected, and, at the same time, the *Lactococcus lactis* NZ3900 containing control pNZ8149 plasmid was processed as a blank control according to the method described above. After sonication, the supernatants were subjected to western blot analysis. The specific steps were as follows: after 12% SDS-PAGE, the samples were electro-transferred to nitrocellulose membranes, and the nitrocellulose membranes were blocked with 5% skimmed milk at room temperature for 2 h. After washing 3 times with PBST, HA mouse monoclonal antibody was added (1:2000), incubated for 1.5 h at room temperature, and washed 3 times with PBST. Goat anti-mouse infrared-labeled secondary antibody was added, incubated at room temperature for 45 min, washed 3 times with PBST, and an Odyssey infrared scanner was used for imaging.

Preparation of a Lytic Mixture of Recombinant *Lactococcus* Expressing Avian Defensin.

Selective and correct expression of a recombinant protein by recombinant *L. lactis* NZ3900 was carried out according to the described experimental method to induce expression of target protein, LAB inducing expression after the first cleaning bacteria with sterile PBS lotion 2–3 times, resuspend bacteria in PBS, the microbial OD600 value was adjusted to 2 OD (concentration of lactic acid bacteria is about 2 × 10^8^ CFU/mL), according to the method described in “Nisin-Controlled Expression, Protein Extraction, and Western Blotting Analysis” by ultrasound break (ultrasonic time is adjusted for 18 min), followed by centrifugation at 12,000 g for 10 min. The supernatant was collected and filtered using a 0.45-m filter, according to the BCA method for absolute quantification of recombinant proteins, and stored at −80 °C for later use.

### Detection of Immunosignal Pathways of J774-Dual™ Reporting Cell Stimulated by Recombinant Avian-Defensin

J774-Dual™ (2.8 × 10^5^ cells/mL) was seeded in 96-well tissue culture plates. Twenty microliters of the prepared recombinant protein was added. Twenty microliters of *L. lactis* NZ3900 lysed supernatant was used as a negative (non-stimulated) control. LPS was used as a positive control. After 24 h of stimulation at 37 °C in an atmosphere containing 5% CO_2_, culture supernatants were collected, clarified by centrifugation, and stored at −20 °C. Then, 170 mL of resuspended QUANTI-Blue™ was added to each well of a flat-bottom 96-well plate. A further 30 mL of J774-Dual™ cell supernatant was added. The plate was incubated at 37 °C in a CO_2_ incubator for 1–8 h. The NF-κB-induced SEAP levels were determined using a microplate spectrophotometer at 650 nm.

J774-Dual™ cells allow simultaneous study of the IRF pathway. J774-Dual™ (2.8 × 10^5^ cells/mL) was seeded in 96-well tissue culture plates. Twenty microliters of the prepared recombinant protein was added. Twenty microliters *L. lactis* NZ3900 lysed supernatant was used as a negative (non-stimulated) control. LPS was used as a positive control. After 24 h of stimulation at 37 °C in an atmosphere containing 5% CO_2_, culture supernatants were collected. The expression of luciferase was detected by microporous plate chemiluminescence detector (LB 960). The following luminometer parameters were set: 50 μL QUANTI-Luc™ injection and end-point measurement with a 4-s start time and 0.1-s reading time. Pipet samples (20 μL per well) were placed in a 96-well white (opaque), black plate, or aluminate tube. The injector was primed with the assay solution before proceeding with the measurements.

### PBMCs and HD11 Stimulated by Recombinant Avian-Defensin

PBMCSs (1 × 10^6^ cells/mL) were seeded in 24-well tissue culture plates. Twenty microliters of the prepared recombinant protein was added. Fifty microliters of *L. lactis* NZ3900 lysed supernatant was used as a negative (non-stimulated) control. Based on preliminary time-course studies, 24 h stimulation is the optimal time period for cytokine responses of bacteria-stimulated PBMCSs. After 24 h of stimulation at 37 °C in an atmosphere containing 5% CO_2_, culture supernatants were collected, clarified by centrifugation, and stored at −20 °C until cytokine analysis. Neither medium acidification nor bacterial proliferation was observed. Quantitative analysis of the IL-12p70 and IL-10 mRNA transcription levels in cells using SYBY Green I quantitative PCR. The HD11 stimulation experiment was identical to the PBMCS experiment.

### Experimental Chickens

Specific-pathogen-free (SPF) chickens were purchased from the HVRI Experimental Animal Center, the CAAS (Harbin, China) and raised in a negative pressure-filtered air isolator. In this study, all animal experiments were approved by the HVRI of CAAS and were carried out in accordance with animal ethics guidelines and approved protocols. SPF chickens were obtained from the Model Animal Research Center of the Harbin Veterinary Research Institute (Harbin, China). All chickens were 10-day-old females that were bred and maintained under specific pathogen-free conditions.

### Using Recombinant rAvBD1-2–6-13 as an Immune Adjuvant for Live Vaccines

The 10-day-old SPF chickens were divided into 4 groups: blank control group, vector control group, vaccine control group, and rAvBD1-2–6-13 adjuvant group. Each group consisted of 10 chickens. First, live Lasota vaccine (1000 doses) was diluted with PBS to 50 mL (50 μL/chicken), and the vaccine control group was injected with 50 μL Lasota strain live vaccine intramuscularly. Each animal in the rAvBD1-2–6-13 adjuvant group was injected 150 μL (diluted 50 μL Lasota strain live vaccine plus 100 μL *L. lactis* NZ3900 lysed supernatant prepared using the above method). In the vector control group, 150 μL (50 μL PBS plus 100 μL wild *Lactococcus* strain lysate) were injected into each muscle, and 150 μL PBS was injected into each muscle of the blank control group. Blood, serum, and anti-coagulant blood were collected every week after immunization, and PBMCs were prepared. At the 3rd week after immunization, 1000 ELD_50_ of F48E9-NDV virulent virus was challenged orally, and blood serum and anti-coagulant blood were collected to prepare PBMCs.

### Using Recombinant rAvBD1-2–6-13 as an Inactivated Vaccine Immune Adjuvant

Preparation of Recombinant β-Defensin rAvBD1-2–6-13 Oil Emulsion: For water phase preparation, we added the recombinant β-defensin rAvBD1-2–6-13 prepared according to the procedure detailed in “Preparation of a Lytic Mixture of Recombinant *Lactococcus* Expressing Avian Defensin” to the prepared surfactant Tween-80 in a 0.25% proportion and incubated at 37 °C for 30 min. The mixture was shaken well and left still for use. The oil phase was prepared by making up a certain ratio of white oil plus aluminum stearate plus Span-80 and sterilized for later use. The emulsion was composed of 2 parts oil-phase (oil emulsion) and 1 part water-phase (recombinant β-defensin rAvBD1-2–6-13 solution with Tween-80), mixed in two phases, and emulsified into W/O type oil emulsion.

Preparation of Recombinant β-Defensin rAvBD1-2–6-13 Immune Adjuvant Newcastle Disease Oil Emulsion Inactivated Vaccine: One part of the chicken Newcastle disease oil emulsion inactivated vaccine was added to 1 part of recombinant β-defensin rAvBD1-2–6-13 oil emulsion, and the mixture was demulsified and re-emulsified by an emulsifier to obtain recombinant β-defensin rAvBD1-2–6-13 immune adjuvant Newcastle disease oil emulsion inactivated vaccine.

The 10-day-old SPF chickens were divided into 4 groups: a blank control group, an empty vector control group, a vaccine control group, and a recombinant β-defensin rAvBD1-2–6-13 adjuvant immunization group, with 10 chickens in each group. In the recombinant β-defensin rAvBD1-2–6-13 immune adjuvant Newcastle disease oil emulsion inactivated vaccine group, the injection dose was 0.6 mL/feather. In the Newcastle disease oil emulsion inactivated vaccine control group, the injection dose was 0.3 mL/feather (The amount of antigen in the immune adjuvant group is the same as that in the vaccine control group). The empty carrier control group was injected intramuscularly with 0.3 mL wild *Lactococcus* strain lysate. The blank control group was injected intramuscularly with 0.3 mL PBS.

After immunization, blood was collected every week, serum was prepared, anti-coagulant blood was collected, and PBMCs were prepared. In the third week after immunization, 1000 ELD_50_ of virulent strains of F48E9-NDV were orally challenged, blood was collected weekly to prepare serum, and anti-coagulant blood was collected to prepare PBMCs.

### Hemagglutination Inhibition Test to Detect Serum Antibody Titer

The Lasota strain NDV virus solution was diluted in a 96-well hemagglutination plate, and 50 μL of the prepared 0.5% chicken red blood cell suspension was added to each well and then placed in a 37 °C incubator for 30 min to determine the HA titer. According to the HA titer of the Lasota strain virus, 8 units and 4 units of virus fluid were prepared. To prepare a 96-well hemagglutination plate, 50 μL of 8-unit virus dilution was added in the first column, and 50 μL of 4-unit virus dilution was added in each of the remaining wells, one serum was tested per row, and 50 μL were added to the first well in the first column for testing. The serum was diluted in multiples, and positive control wells and negative control wells were set at the same time. After that, 50 μL of prepared 0.5% chicken red blood cell suspension was added to all wells and then placed in a 37 °C incubator for 30 min for interpretation. The antibody titer is expressed as 1:2^*n*^ (*n* is the number of wells with the highest dilution times of the serum whose erythrocyte agglutination is 100% inhibited) or as nLog2 in a negative logarithm of 1:2^*n*^.

### Serum ELISA Antibody Test

Blood was collected 1 week after immunization, and the serum was separated. Chicken IL-1β, IL-4, IL-10, and IFN-γ concentrations in the culture medium were measured by sandwich ELISA using commercially available chicken ELISA kits (Solarbio, China) following the manufacturer’s instructions.

### PBMC Proliferation Activity Assay

PBMC proliferation was assessed by the CCK-8 method. The immunized chickens were euthanized at weeks 3 and 4. PBMCs were isolated using a chicken peripheral blood lymphocyte separation solution kit and washed twice in fresh RPMI 1640 medium. The cells were resuspended at 1 × 10^6^ cells/mL in RPMI 1640 medium with 10% fetal bovine serum (FBS), 100 units/mL penicillin, 100 μg/mL streptomycin, 100 μg/mL Normocin, 100 μg/mL gentamicin, and 100 μg/mL bacitracin. We added 100 μL cells to 96-well plates and stimulated in vitro for 48 h at 37 °C in a 5% CO_2_ incubator with either concanavalin A (ConA, 5 μg/mL, Sigma) and 2-acetoxy-1-methoxypropane (PMA, 1 μg/mL, Sigma) mixture as an irritant. Then, 100 μL cells was added to 96-well plates and stimulated in vitro for 72 h at 37 °C in a 5% CO_2_ incubator with lipopolysaccharide (LPS, 1 μg/mL, Sigma) as an irritant. An untreated culture served as a negative control. Ten microliters of CCK-8 was then added to each well, incubation was continued for 4 h, and the absorbance at 450 nm was determined using a microplate reader.

### Statistical Analysis of Data

Statistical analyses were carried out using ordinary one-way ANOVA to evaluate statistical differences among groups, and *P* values of 0.05 were considered statistically significant. *P* < 0.01 was considered highly significant, *P* < 0.001 was considered very highly significant, and *P* < 0.0001 was considered extremely highly significant.

## Results

Construction of Recombinant *Lactococcus* and Expression of the Target Protein.

**Fig. 1:**
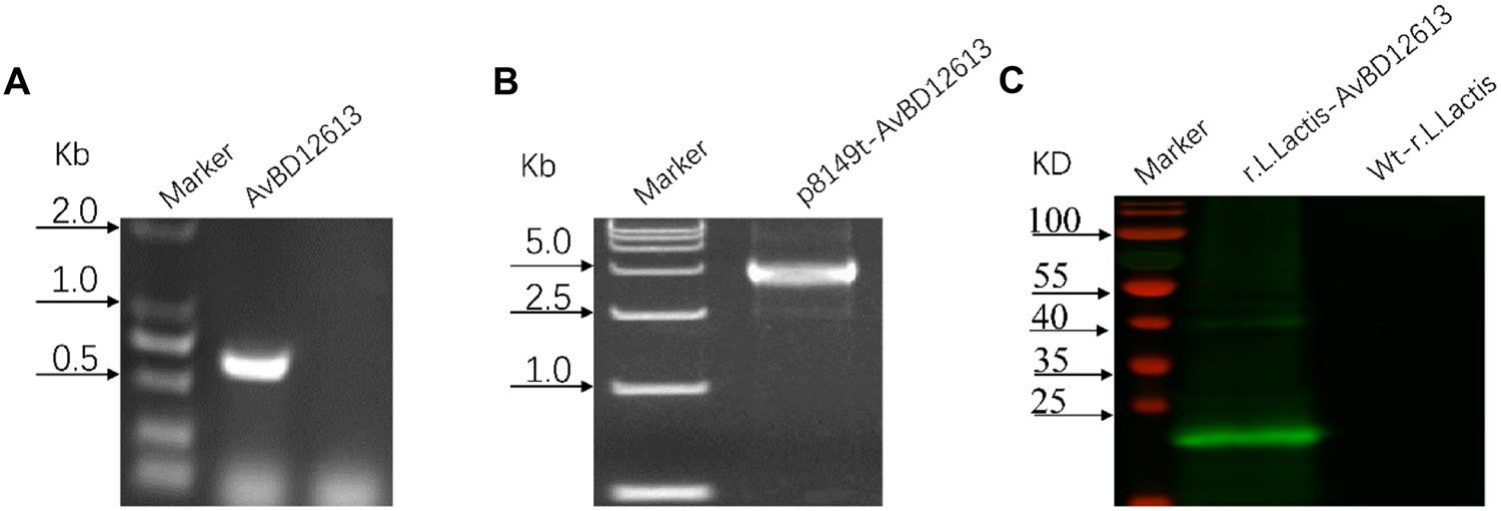
Construction of the plasmid pNZ8149t-AvBD1-2–6-13 expressing the AvBD1-2–6-13 fusion protein. **A** PCR amplification of Opti-VP2 optimization gene (675 bp); **B** the recombination plasmid pNZ8149t-AvBD1-2–6-13 was linearized with NcoI to obtain a 5295 bp fragment. **C** Identification of recombinant proteins via western blotting analysis. Immunoblot analysis of total whole-cell protein extracts from recombinant r-*L. lactis*-AvBD1-2–6-13 (lane 1) and wt-*L. lactis* (lane 2)

First, we amplified the rAvBD1-2–6-13 fusion gene fragment of 657 bp (Fig. 1A, lane 2), recombined the AvBD1-2–6-13 fusion gene fragment with the pNZ8149 linear vector, and transferred it to *L. lactis* NZ3900. Following extraction, the plasmid was identified by *Nco*I digestion. Linear fragments of the expected size (5295 bp) were obtained (Fig. 1B, lane 2). After protein expression was induced in recombinant *L. lactis* NZ3900 expression, the recombinant *L. lactis* NZ3900 were sonicated and the rAvBD1-2–6-13 protein (approximately 35 kDa) matching the expected target protein size was identified by western blotting; the pNZ8149 empty vector strain was used as a negative control (Fig. 1C, lanes 2 and 3). The concentration of rAvBD1-2–6-13 protein detected by the BCA method was 27 μg/mL.

### Detection of IRF and NF-κB Signaling Pathways Activated by Recombinant β-Defensin inJ774-Dual™ Cells

**Fig. 2:**
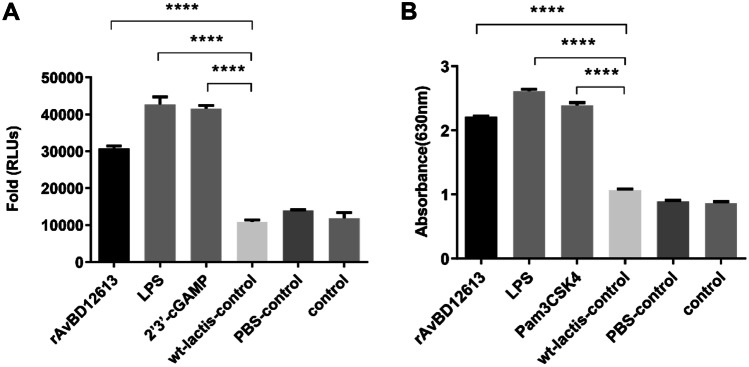
Recombinant β-defensin rAvBD1-2–6-13 can efficiently activate the IRF interferon regulatory factor signaling pathway of macrophage J774-Dual™ cells. **A** Detection of IRF signaling pathways activated by recombinant β-defensin rAvBD1-2–6-13 in J774-Dual™ cells, positive standard control group stimulated by LPS or 2′3′-cGAMP. **B** Detection of NF-κB signaling pathways activated by recombinant β-defensin rAvBD1-2–6-13 in J774-Dual™ cells, positive standard control group stimulated by LPS or Pam3CSK4. Data are presented as mean ± SD. ****P < 0.0001

The results of our studies showed that recombinant β-defensin rAvBD1-2–6-13 can efficiently activate the interferon regulatory factor (IRF) signaling pathway in macrophage J774-Dual™ cells. Compared with the wild bacteria control group, negative blank control group, and PBS control group, the positive control group stimulated by LPS or 2′, 3′-cGAMP has differences extremely significant (*P* < 0.0001; Fig. 2A); the recombinant β-defensin rAvBD1-2–6-13 was also extremely significantly higher than the wild bacteria control group, negative blank control group, and PBS control group (*P* < 0.0001; Fig. 2A).

Recombinant β-defensin rAvBD1-2–6-13 can also efficiently activate the NF-κB signaling pathway of macrophage J774-Dual™ cells. Compared with the wild bacteria control group, negative blank control group, and PBS control group, the positive control group stimulated by LPS or 2′,3′-cGAMP has differences extremely significant (*P* < 0.0001; Fig. 2B); the recombinant β-defensin rAvBD1-2–6-13 group was also extremely significantly higher than the wild bacteria control group, negative blank control group, and PBS control group (*P* < 0.0001; Fig. 2B).

### Detection of Chicken PBMCs Activated by Recombinant β-Defensins

**Fig. 3:**
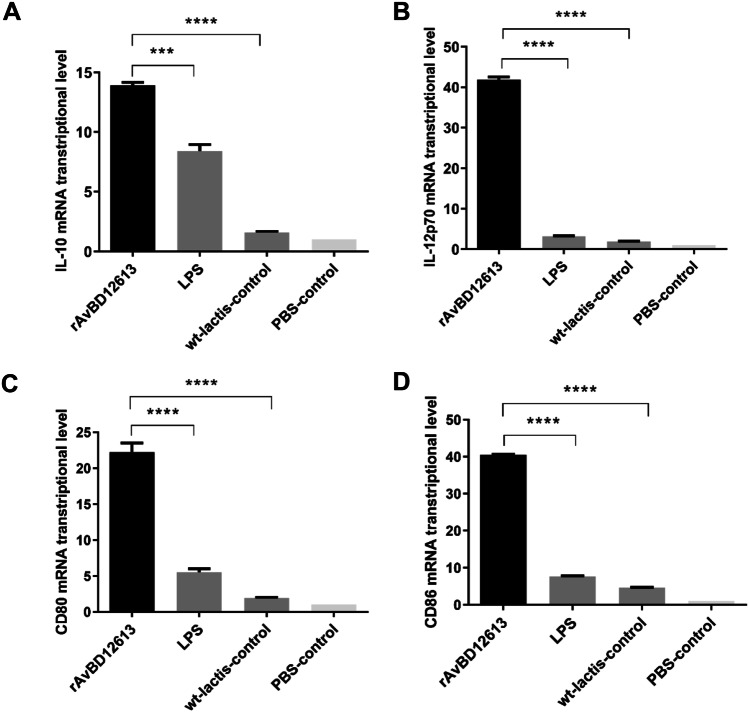
Detection results of cytokine mRNA transcription levels and co-stimulatory factor expression after rAvBD1-2–6-13 stimulation of chicken PBMCs, positive standard control group stimulated by LPS. **A** IL-10, **B** IL-12p70, **C** CD80, **D** CD86. Data are presented as mean ± SD. ***P < 0.001; ****P < 0.0001

Recombinant β-defensin rAvBD1-2–6-13 efficiently stimulated chicken PBMCs to produce cytokine IL-10 (Fig. 3A). The IL-10 production in the recombinant β-defensin rAvBD1-2–6-13 group was significantly enhanced compared with the wild bacteria control group (12.3-fold increase) and PBS control group (13.9-fold increase) (*P* < 0.0001; Fig. 3A). Compared with the LPS-stimulated chicken PBMC control group (5.41-fold increase), the difference was extremely significant (*P* < 0.001; Fig. 3A). Furthermore, the results of IL-12 detection showed that recombinant β-defensins rAvBD1-2–6-13 efficiently stimulated the production of cytokine IL-12 from chicken PBMCs (*P* < 0.0001; Fig. 3B). Compared with the wild bacteria control group (39.96-fold increase), PBS control group (41.87-fold increase), and LPS-stimulated chicken PBMCs control group (38.70-fold increase), the difference was extremely significant (*P* < 0.0001), even higher than the LPS positive control group (Fig. 3B). The results showed that the recombinant protein rAvBD1-2–6-13 effectively activated chicken PBMCs, which upregulated the expression of IL-12 for immune activation and of IL-10 for negative immune regulation to maintain immune homeostasis.

Furthermore, our results showed that recombinant β-defensin rAvBD1-2–6-13 significantly increased the expression of CD80 and CD86 in chicken PBMCs (Fig. 3C, D). Treatment with recombinant β-defensin rAvBD1-2–6-13 significantly upregulated the expression of CD80 and CD86 compared with the wild bacteria control group (20.31- and 35.89-fold increase, respectively), LPS-stimulated chicken PBMCs positive control group (16.74- and 32.91-fold increase, respectively), and PBS control group (22.24- and 40.51-fold increase, respectively) (*P* < 0.0001; Fig. 3C, D). Collectively, these results showed that recombinant β-defensin rAvBD1-2–6-13 can significantly activate chicken PBMCs and activate cellular immune responses.

### Detection of HD11 Activation by Recombinant β-Defensin

**Fig. 4:**
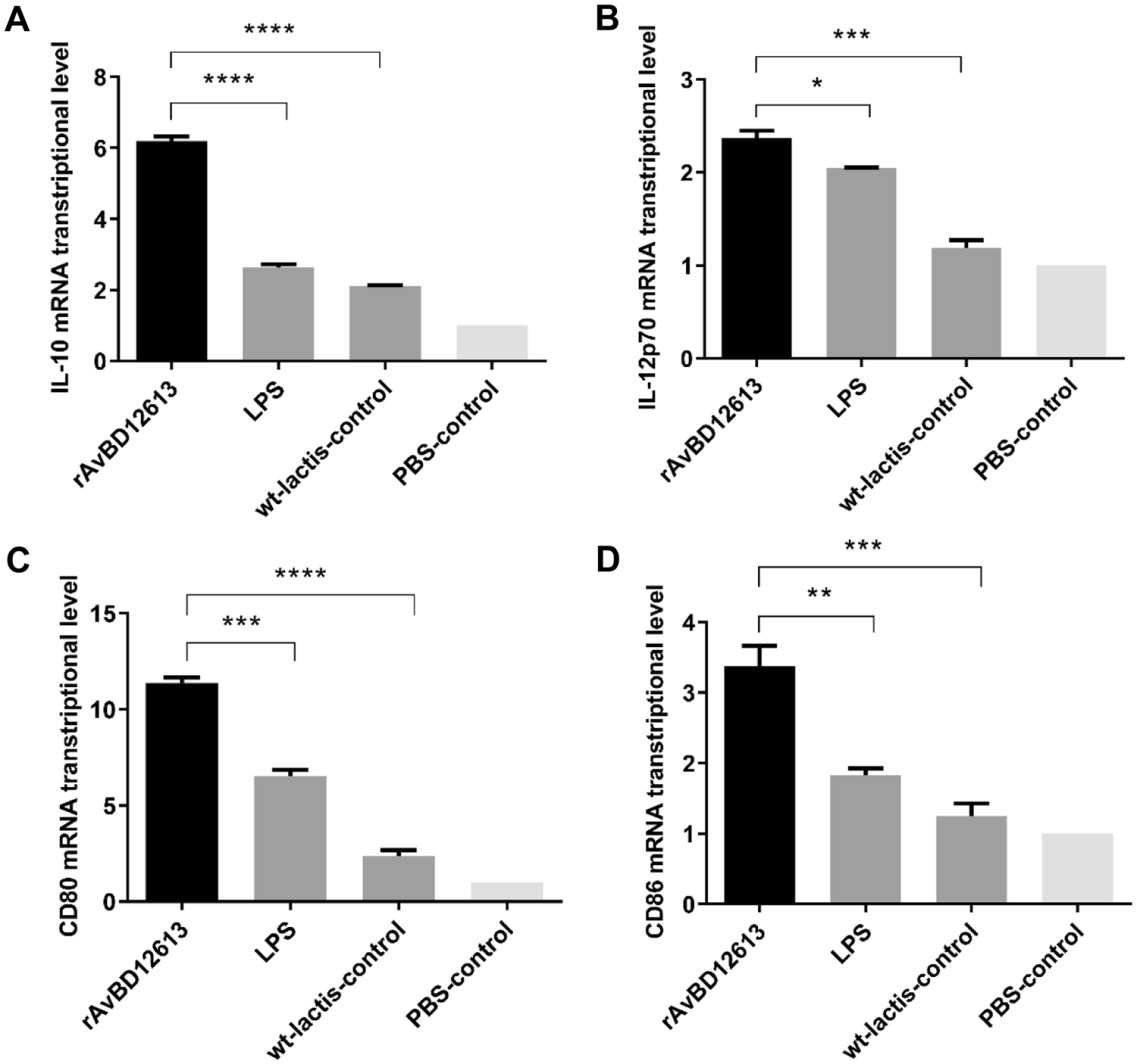
Detection results of cytokine mRNA transcription levels and co-stimulatory factor expression after rAvBD1-2–6-13 stimulation of chicken HD11 cells, positive standard control group stimulated by LPS. **A** IL-10, **B** IL-12p70, **C** CD80, **D** CD86. Data are presented as mean ± SD. **P < 0.01; ***P < 0.001; ****P < 0.0001

The results showed that recombinant β-defensins rAvBD1-2–6-13 can significantly increase the expression of IL-10 in chicken HD11 cells. Compared with the wild bacteria control group (4.08-fold increase) and PBS control group (6.19-fold increase), the recombinant β-defensin rAvBD1-2–6-13 group has extremely significant differences (*P* < 0.0001; Fig. 4A). Compared with the LPS-stimulated chicken HD11 positive standard control group, it is significantly (3.35-fold increase) higher than the LPS positive control group, the difference is extremely significant (*P* < 0.001; Fig. 4A). Recombinant β-defensins rAvBD1-2–6-13 significantly increased the expression of IL-12 in chicken HD11 cells (Fig. 4B) compared with the wild bacteria control group (1.18-fold increase) and the PBS control group (2.37-fold increase), the recombinant β-defensin rAvBD1-2–6-13 group has extremely significant differences (*P* < 0.001; Fig. 4B). Compared with the LPS-stimulated chicken HD11 positive standard control group (0.32-fold increase), it is significantly different (*P* < 0.05; Fig. 4B). The results showed that recombinant β-defensin rAvBD1-2–6-13 significantly activated HD11 and produced immunomodulatory cytokines, such as IL-10 and IL-12.

The results also showed that recombinant β-defensin rAvBD1-2–6-13 could significantly increase the expression of CD80 and CD86 in chicken HD11 (Fig. 4C, D). Compared with the wild bacteria control group (9- and 2.13-fold increase, respectively), and the PBS control group (11.37- and 3.38-fold increase, respectively), the recombinant β-defensin rAvBD1-2–6-13 group showed extremely significant differences (*P* < 0.0001; Fig. 4C, D); compared with the LPS-stimulated chicken HD11 positive standard control group (4.84- and 1.55-fold increase, respectively), it was significantly higher (*P* < 0.001; Fig. 4C, D). The results showed that recombinant β-defensin rAvBD1-2–6-13 could significantly activate HD11 to express CD80 and CD86 molecules.

### HI Test to Detect Serum Antibody Titer

**Fig. 5:**
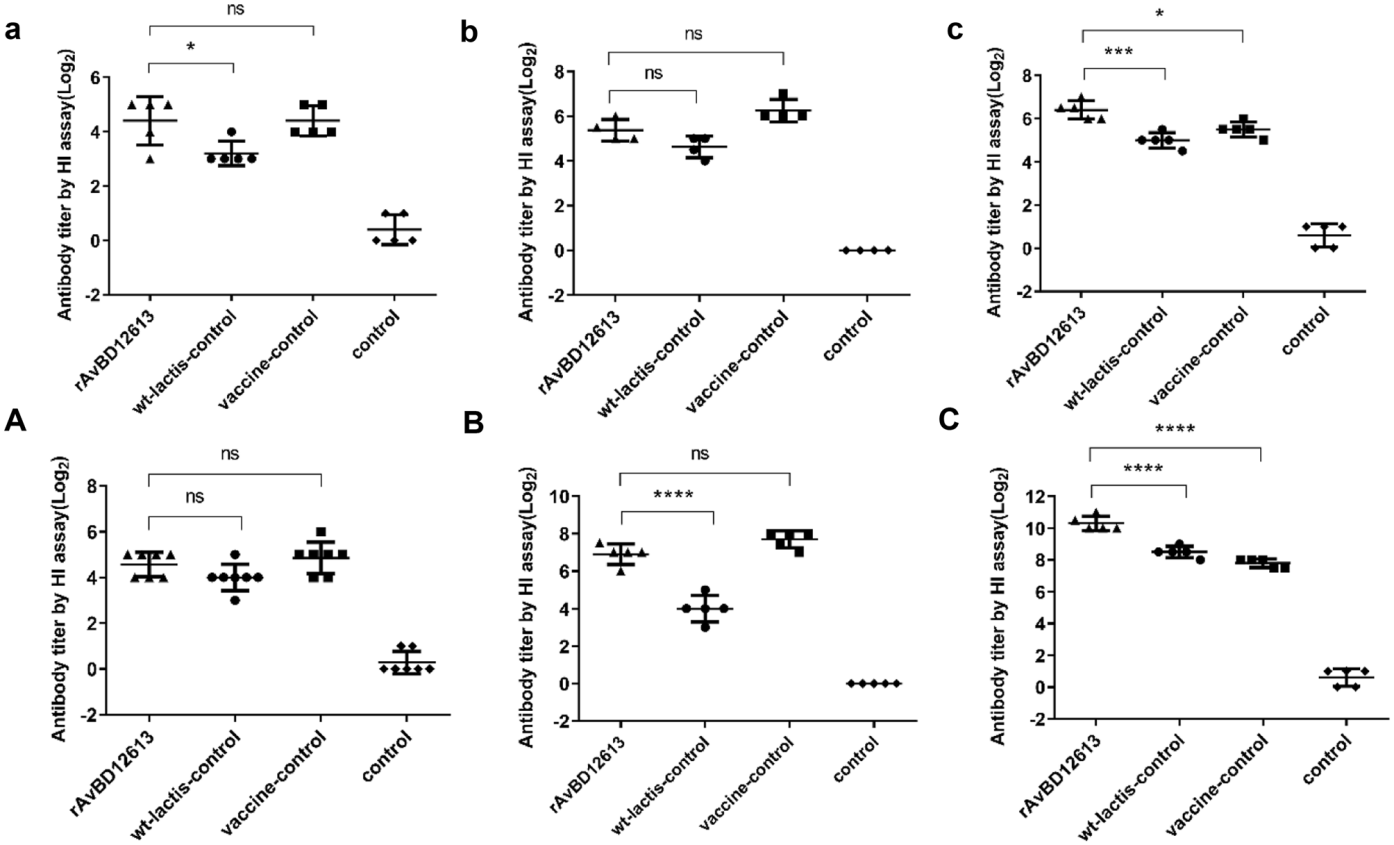
Titers of antibodies against NDV in the sera of chickens obtained via HI test. In the animal experiment of r-*L. lactis*-AvBD1-2–6-13 as the adjuvant of the live Newcastle disease vaccine, titers of antibodies against NDV after immunization in (**a**) the second week, (**b**) the third week, and (**c**) the fourth week (after 3 weeks of immunization and 1 week of the challenge). In the animal experiment of r-*L. lactis*-AvBD1-2–6-13 as the adjuvant of the inactivated Newcastle disease vaccine, titers of antibodies against NDV after immunization in (**A**) the second week, (**B**) the third week, and (**C**) the fourth week (after 3 weeks of immunization and 1 week of the challenge). Data are presented as mean ± SD. ^ns^*P* > 0.05; *P < 0.05; **P < 0.01; ***P < 0.001; ****P < 0.0001

In the animal, r-*L. lactis*-AvBD1-2–6-13 was used as an adjuvant for the live Newcastle disease vaccine and the ND antibody titer of the recombinant β-defensin rAvBD1-2–6-13 immune adjuvant group was compared with that of live vaccine control group (no adjuvant) at 2 and 3 weeks after immunization. Our results showed that the difference was not significant at 2 and 3 weeks after immunization (*P* > 0.05; Fig. 5a, b); however, after 3 weeks of immunization and 1 week of challenge, the ND antibody titer of the recombinant β-defensin rAvBD1-2–6-13 adjuvant group was significantly higher than that of the vaccine control group (no adjuvant vaccine control group) (*P* < 0.05; Fig. 5c). The results showed that the recombinant β-defensin rAvBD1-2–6-13 immune adjuvant could significantly improve the anti-NDV activity of chickens.

In another set of animal experiment, r-*L. lactis*-AvBD1-2–6-13 was used as an adjuvant for the inactivated vaccine against Newcastle disease and the ND antibody titer of the recombinant β-defensin rAvBD1-2–6-13 immune adjuvant group was compared with the vaccine control group (no adjuvant vaccine) at 2 and 3 weeks after immunization. The difference was not significant (*P* > 0.05; Fig. 5A, B), but after 3 weeks of immunization and 1 week of the challenge, the ND antibody titer of the recombinant β-defensin rAvBD1-2–6-13 immune adjuvant group was extremely significantly higher than that of the vaccine control group (no adjuvant vaccine control group) (*P* < 0.0001; Fig. 5C). The results showed that the recombinant β-defensin rAvBD1-2–6-13 immune adjuvant could significantly improve chicken anti-NDV activity, especially anti-NDV activity during a viral attack.

### Detection of Serum Cytokine by ELISA

**Fig. 6:**
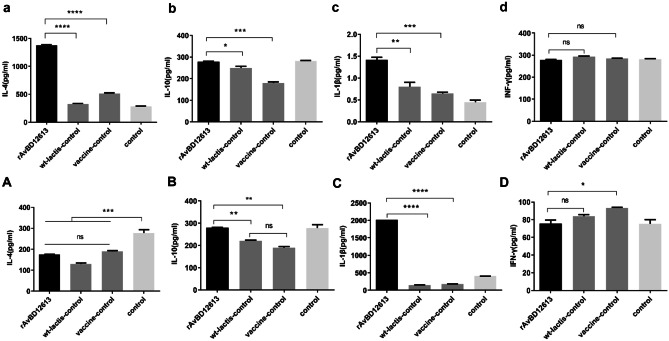
Detection of serum cytokine after one week of immunization by ELISA. The concentration of (**a**) IL-4, (**b**) IL-10, (**c**) IL-1β, (**d**) IFN-γ in the animal experiment of r*-L. lactis*-AvBD1-2–6-13 as the adjuvant of live vaccine. The concentration of (**A**) IL-4, (**B**) IL-10, (**C**) IL-1β, (**D**) IFN-γ in the animal experiment of r-*L. lactis*-AvBD1-2–6-13 as the adjuvant of inactivated vaccine. Data are presented as mean ± SD. ^ns^*P* > 0.05; *P < 0.05; **P < 0.01; ***P < 0.001; ****P < 0.0001

In the animal experiment using r-*L. lactis*-AvBD1-2–6-13 as a live vaccine adjuvant, the concentrations of IL-4, IL-10, and IL-1β in the recombinant β-defensin rAvBD1-2–6-13 immune adjuvant group were extremely significantly higher than those in the vaccine control group (without adjuvant) (*P* < 0.0001; Fig. 6a, b, c). Compared with the recombinant β-defensin rAvBD1-2–6-13 immune adjuvant group and the non-adjuvant vaccine group, the concentration of IFN-γ was not significantly different (*P* > 0.05; Fig. 6d).

In the animal experiment using r-*L. lactis*-AvBD1-2–6-13 as an adjuvant for the Newcastle disease inactivated vaccine, after 1 week of immunization, the recombinant β-defensin rAvBD1-2–6-13 immune adjuvant group and the non-adjuvant vaccine group showed no significant difference in IL-4 concentration (*P* > 0.05; Fig. 6A). The concentration of IL-10 was significantly higher than that of the unadjuvanted vaccine control group (*P* < 0.01; Fig. 6B), and the concentration of IL-1β was extremely significantly higher than that of the unadjuvanted vaccine control group (*P* < 0.0001; Fig. 6C). The concentration of IFN-γ was significantly lower than that of the unadjuvanted vaccine control group (*P* < 0.05; Fig. 6D). At the same time, the concentrations of IL-10 and IL-1β increased, indicating that activation of the immune response did not cause inflammation.

### Detection of Cell Proliferation Activity of Chicken PBMCs

**Fig. 7:**
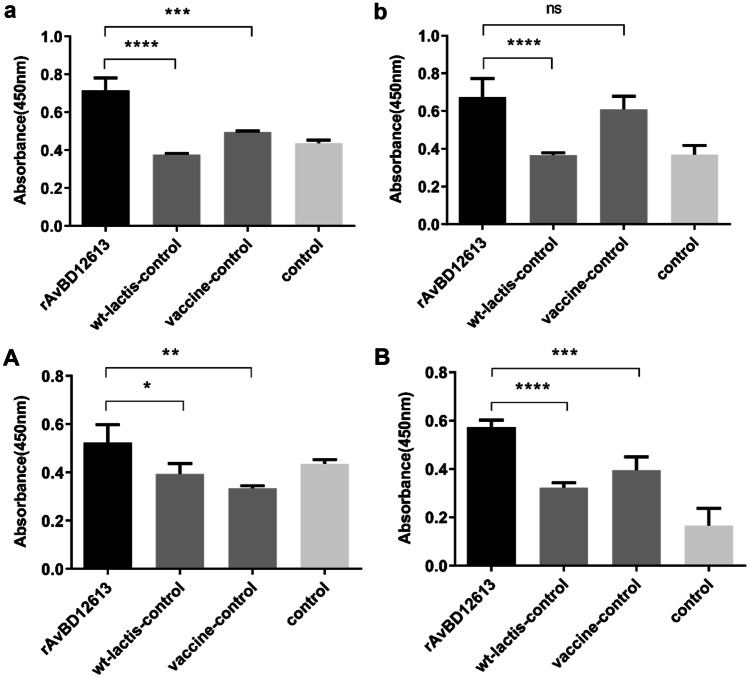
Detection of cell proliferation activity of chicken PBMCs by LPS stimulation. In the animal experiment of r-*L. lactis*-AvBD1-2–6-13 as the adjuvant of the live Newcastle disease vaccine, proliferation activity after immunization in (**a**) the third week and (**b**) the fourth week (after 3 weeks of immunization and 1 week of the challenge). In the animal experiment of r-*L. lactis*-AvBD1-2–6-13 as the adjuvant of the inactivated Newcastle disease vaccine, proliferation activity after immunization in (**A**) the third week and (**B**) the fourth week (after 3 weeks of immunization and 1 week of the challenge). Data are presented as mean ± SD. ^ns^*P* > 0.05; *P < 0.05; **P < 0.01; ***P < 0.001; ****P < 0.0001

In the animal experiment using r-*L. lactis*-AvBD1-2–6-13 as the adjuvant of the live Newcastle disease vaccine, the proliferation activity of PBMCs in the recombinant β-defensin rAvBD1-2–6-13 immune adjuvant group (activated by LPS) was extremely higher than that of the adjuvant-free vaccine control group (*P* < 0.001; Fig. 7a). After 3 weeks of immunization and 1 week after challenge, the proliferation activity of PBMCs in the recombinant β-defensin rAvBD1-2–6-13 immunoadjuvant group was compared with that of the adjuvant-free vaccine control group. The difference was not significant (*P* > 0.05; Fig. 7b), but extremely higher than the wt-*L. lactis* control group (*P* < 0.001; Fig. 7b).

When using r-*L. lactis*-AvBD1-2–6-13 as an adjuvant for the Newcastle disease inactivated vaccine, after 3 weeks of immunization, the proliferation activity of PBMCs in the recombinant β-defensin rAvBD1-2–6-13 adjuvant group (activated by LPS) was higher than that of the adjuvant-free vaccine control group (*P* < 0.01; Fig. 7A). After 3 weeks of immunization and 1 week after challenge, the proliferation activity of PBMCs in the recombinant β-defensin rAvBD1-2–6-13 immunoadjuvant group was extremely significantly higher than that of the adjuvant-free vaccine control group (*P* < 0.001; Fig. 7B). The results showed that the recombinant β-defensin rAvBD1-2–6-13 immune adjuvant could significantly activate antigen-presenting cells of immunized chicken PBMCs.

**Fig. 8:**
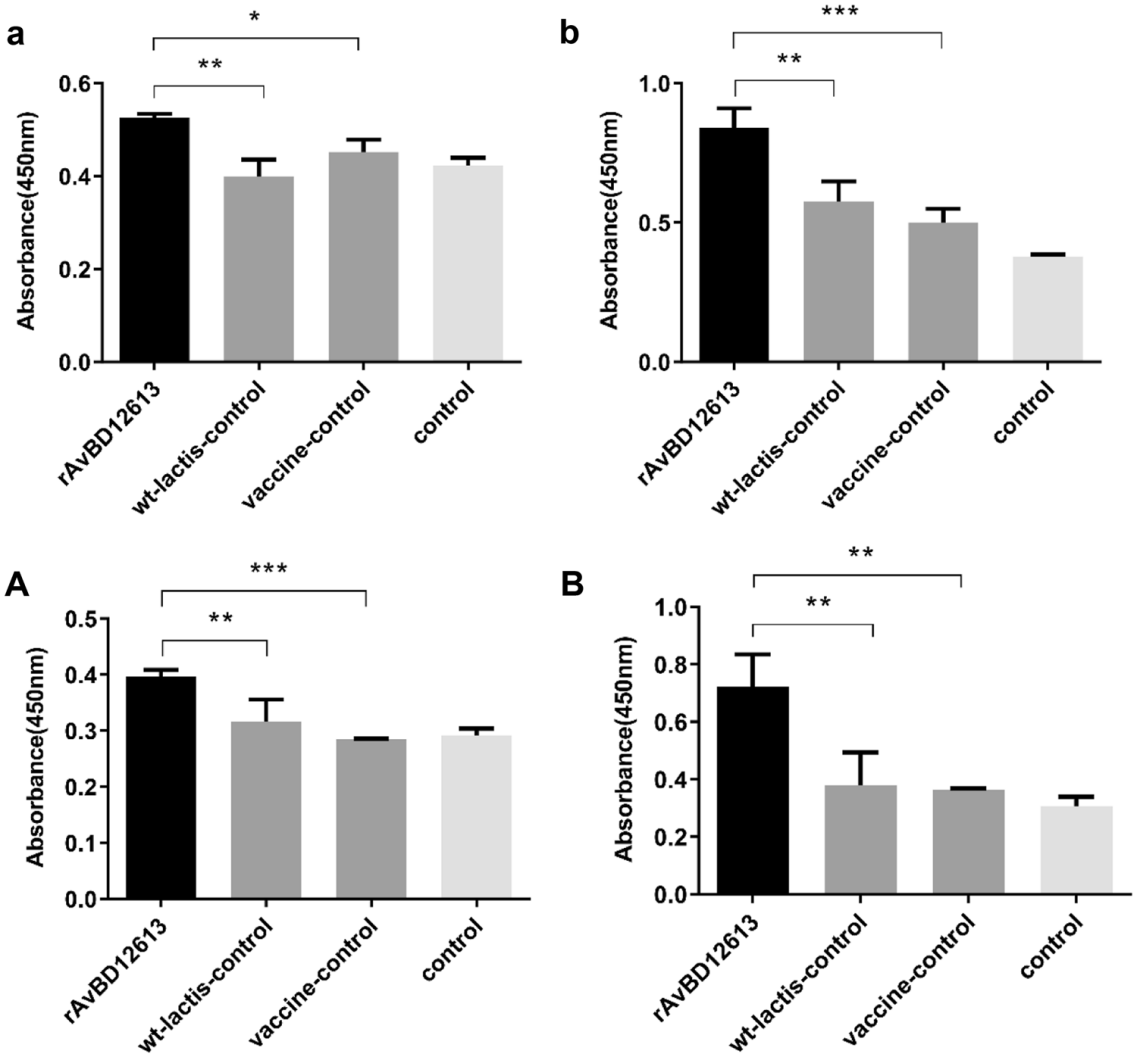
Detection of cell proliferation activity of chicken PBMCs activated by ConA and PMA. In the animal experiment of r-*L. lactis*-AvBD1-2–6-13 as the adjuvant of the live Newcastle disease vaccine, proliferation activity after immunization in (**a**) the third week and (**b**) the fourth week (after 3 weeks of immunization and 1 week of the challenge). In the animal experiment of r-*L. lactis*-AvBD1-2–6-13 as the adjuvant of the inactivated Newcastle disease vaccine, proliferation activity after immunization in (**A**) the third week and (**B**) the fourth week (after 3 weeks of immunization and 1 week of the challenge). Data are presented as mean ± SD. *P < 0.05; **P < 0.01; ***P < 0.001

When r-*L. lactis*-AvBD1-2–6-13 was used as an adjuvant for the live Newcastle disease vaccine, after 3 weeks of immunization, the proliferation activity of PBMCs in the recombinant β-defensin rAvBD1-2–6-13 adjuvant group (activated by ConA and PMA) was significantly higher than that of the adjuvant-free vaccine control group (*P* < 0.01; Fig. 8a). After 3 weeks of immunization and 1 week after challenge, the proliferation activity of PBMCs in the recombinant β-defensin rAvBD1-2–6-13 adjuvant group was extremely significantly higher than that in the adjuvant-free vaccine control group (*P* < 0.001; Fig. 8b).

Using r-*L. lactis*-AvBD1-2–6-13 as an adjuvant for the inactivated Newcastle disease vaccine, after 3 weeks of immunization, the proliferation activity of PBMCs in the recombinant β-defensin rAvBD1-2–6-13 adjuvant group (activated by ConA and PMA) was extremely significantly higher than that of the adjuvant-free vaccine control group (*P* < 0.001; Fig. 8A). After 3 weeks of immunization and 1 week of challenge, the proliferation activity of PBMCs in the recombinant β-defensin rAvBD1-2–6-13 immune adjuvant group was significantly higher than that of the non-adjuvant vaccine control group (*P* < 0.01; Fig. 8B). The results showed that the recombinant β-defensin rAvBD1-2–6-13 immune adjuvant could significantly activate the T cells of the peripheral blood mononuclear cells of immunized chickens and improve the immune response.

## Discussion

The protein expression system is composed of a host, foreign genes, vectors, and auxiliary components. Through this system, expressing foreign genes in the host can be achieved. The composition and structure of different expression systems are different, and the amount of protein expression, expression efficiency, and biological function will be different. To ensure the correct expression of avian β-defensin, a food-grade nisin-inducible expression system was used in this study. The food safety-grade host strain (food-grade *Lactococcus lactis* NZ3900 strain) was used as the expression host strain. The food-grade *Lactococcus lactis* NZ3900 strain evolved from probiotic strains that have been used in humans and animals for thousands of years and are currently recognized as food grade probiotics. The induction expression system used the food-grade inducer nisin is also recognized as a natural anti-bacterial peptide that has been used for hundreds of years. Therefore, the recombinant *L. lactis* NZ3900 constructed in this study and the recombinant β-defensins expressed in this study fully meet the requirements of biosafety and green food safety.

This study found that recombinant β-defensin rAvBD1-2–6-13 could induce immune cells to produce higher levels of IL-12 and IFN-γ in vivo and in vitro and, at the same time, induce higher levels of IL-10. IL-12 and IFN-γ are typical Th1 type cytokines. Their main function is to promote the immune response, promote the development and differentiation of lymphocytes and macrophages, and at the same time inhibit the secretion of Treg-type cytokines, and IL-10 is a typical Treg type cytokine. Its main function is to inhibit the Th1 type immune response and the production and release of inflammatory cytokines and to carry out negative immune regulation. Modern immunology shows that the biological effects of IL-10 are surprisingly multifaceted and dual-faceted. The target cells of IL-10 inhibitory action include lymphocytes, monocytes, macrophages, mast cells, neutrophils, and eosinophils, and the release of inflammatory mediators and the Th1 immune response. At the same time, IL-10 can promote antigen presentation, and it has an immunostimulatory effect. Therefore, IL-10 is an anti-inflammatory factor with an important immunomodulatory effect on the body [21, 22]. The recombinant β-defensin rAvBD1-2–6-13 in this study can be used as an immune adjuvant to activate the immune response while maintaining immune regulation at the same time.

In this study, recombinant β-defensin rAvBD1-2–6-13 was used as an immune adjuvant to induce the body to produce higher levels of cytokine IL-4 and pro-inflammatory cytokine IL-1β. IL-4 is a TH2 cytokine, which is mainly produced by activated T cells. It is a multifunctional cytokine that plays an important role in the immune system. IL-4 can activate T cells, B cells, and macrophages. IL-4 is a chemokine for macrophages that promotes antigen presentation by macrophages and inhibits the production of monocyte inflammatory cytokines IL-1, TNF, and IL-6. IL-1β is a pro-inflammatory cytokine produced by monocytes and macrophages. It can promote the production of IL-4. A local low concentration of IL-1β can synergistically stimulate APC and T cell activation and promote B cell proliferation and secretion of antibodies [23, 24]. In this study, recombinant β-defensin rAvBD1-2–6-13 was used as an immune adjuvant, which could simultaneously induce the body to produce higher levels of anti-inflammatory factor IL-4 and pro-inflammatory cytokine IL-1β, indicating that injection of recombinant β-defensin rAvBD1-2–6-13 immune adjuvant could activate the body’s immune response while maintaining immune regulation homeostasis. This is consistent with the modern concept of moderate immune regulation. This further demonstrates that recombinant β-defensin rAvBD1-2–6-13 as an immune adjuvant can improve immune response and immunity without strong inflammatory properties and excessive activation of the body’s immune system.

T cell activation requires costimulatory signals between the surface molecules of antigen-presenting cells (APC) and T cells. The two most important cell costimulatory molecules on APC are CD80 (B7-1) and CD86 (B7-2). They all belong to the B7 family. To exert their costimulatory activity, CD80 and CD86 bind to the receptor CD28 on T cells to trigger the activation of T cells and the production of IL-2, which is a key cytokine that induces T cell proliferation. IL-2 also plays a variety of biological roles in other immune cells, including NK cells, B cells, and monocytes [25–31]. In this study, recombinant β-defensin rAvBD1-2–6-13 induced higher levels of CD80 and CD86 in immune cells in vivo and in vitro, indicating that recombinant β-defensin rAvBD1-2–6-13 as an adjuvant can help activate APC and T cells in antigen-presenting cells, thereby enhancing the immune response of the vaccine.

## Data Availability

All raw data and biological material are saved in the State Key Laboratory of Veterinary Biotechnology, Harbin Veterinary Research Institute, Chinese Academy of Agricultural Sciences, Harbin 150,069, China. In case of requirement, please contact the corresponding author for any detailed question.
